# A Case Report of Primary Aldosteronism and Extensive Hypertension-Mediated Organ Damage

**DOI:** 10.7759/cureus.53818

**Published:** 2024-02-08

**Authors:** Elisabete Brum de Sousa, Maria do Mar Menezes, Ana Maria Cordeiro

**Affiliations:** 1 Internal Medicine, Hospital de Santo António dos Capuchos, Centro Hospitalar Universitário Lisboa Central, Lisboa, PRT; 2 Nephrology, Hospital Curry Cabral, Centro Hospitalar Universitário Lisboa Central, Lisboa, PRT; 3 Radiology, Hospital de Santo António dos Capuchos, Centro Hospitalar Universitário Lisboa Central, Lisboa, PRT

**Keywords:** hypertensive retinopathy, target organ damage, hemorragic stroke, end-stage renal disease, primary aldosteronism, secondary hypertension

## Abstract

Primary aldosteronism, the most common curable form of secondary hypertension, is associated with greater hypertension-related organ damage and cardiovascular complications compared to primary essential hypertension.

The authors present a case involving a 41-year-old Black male admitted to the emergency department with left hemiparesis and blurred vision persisting for one hour, accompanied by markedly elevated blood pressure (220/140 mmHg). The patient was asymptomatic by then, and, aside from a history of tobacco smoking and occasional cannabis use, lacked significant medical comorbidities. Further investigations revealed a right acute hemorrhagic stroke, bilateral grade 4 hypertensive retinopathy, chronic kidney disease with end-stage renal disease, hypokalemia, and an elevated aldosterone/renin ratio. An abdominal CT scan showed bilateral adrenal hyperplasia. The patient was diagnosed with primary aldosteronism with extensive hypertension-mediated organ damage.

This case highlights the significant harm caused by undiagnosed primary aldosteronism-induced secondary hypertension, emphasizing the importance of timely diagnosis and intervention to prevent organ damage.

## Introduction

Primary Aldosteronism (PA) is the predominant etiology of secondary arterial hypertension [1,2]. Patients with PA have an augmented incidence of hypertension-mediated organ damage (HMOD) and higher cardiovascular complications than age and sex-matched patients with essential hypertension and the same degree of blood pressure elevation [3-5]. The main causes of PA are adrenal adenomas, unilateral and bilateral adrenal cortex hyperplasia, familial forms of the disease, and aldosterone-secreting adrenal carcinomas [1,2].

The distinctive features of PA involve diminished levels of plasma renin and heightened levels of plasma aldosterone, which are inappropriately elevated considering the individual's volume and blood pressure status [1-3]. Furthermore, PA exerts deleterious effects on the heart, arterial walls, and kidneys [3].

Despite its common occurrence, PA is often underdiagnosed due to its clinical resemblance to primary hypertension [3-5]. Recognizing the underlying cause of PA assumes paramount importance, as unilateral forms can be effectively addressed through adrenalectomy, while bilateral disease necessitates a conservative approach involving aldosterone receptor antagonists [3-5].

## Case presentation

A 41-year-old Black male presented to our emergency department with symptoms of left hemiparesis and blurred vision persisting for one hour. On examination, he displayed right conjugated eye deviation, left homonymous hemianopsia, ipsilateral central facial paresis, upper limb plegia, and lower limb paresis. Blood pressure measurement revealed 220/140 mmHg and the electrocardiogram demonstrated sinus rhythm with a heart rate of 75 beats per minute. The patient was a native of Mozambique, and his medical history was unremarkable, aside from a history of tobacco smoking and occasional cannabis use, with no significant family history reported. He was asymptomatic by then. 

A brain computed tomography (CT) scan was conducted, revealing a spontaneous right nucleobasal hematoma. Subsequent brain CT angiography exhibited no vascular abnormalities.

Laboratory findings included normocytic normochromic anemia (Hb 10.7 g/dL), hypokalemia (2.9 mmol/L), and kidney injury (creatinine 7.32 mg/dL, urea 132 mg/dL). Urinary analysis showed proteinuria without hematuria. The urine protein-creatinine ratio was 324mg/g and the urine albumin-creatinine ratio was 116mg/g.

Furthermore, a kidney ultrasound disclosed signs of chronic kidney disease, presenting with a bipolar diameter of approximately 9.7 cm, increased cortical echogenicity, and reduced differentiation of the central parenchyma.

The patient was admitted to the intensive care unit (ICU) for two days, with the primary goal of controlling and monitoring blood pressure, neurologic deficits, and renal function. The patient's blood pressure was managed initially with intravenous labetalol, followed by oral administration of nifedipine (120 mg/day) and clonidine (450 mcg/day). Neurologic deficits improved, and hypokalemia was corrected with intravenous potassium chloride. Adequate urine output obviated the need for dialysis, enabling the patient's transfer to a medical ward.

A comprehensive diagnostic evaluation was conducted to ascertain the causes of secondary hypertension. Hyporeninemic hyperaldosteronism was identified, characterized by a plasma aldosterone concentration (PAC) of 67.40 ng/dL (normal values: 2.21-35.3 ng/dL), a plasma renin concentration of less than 1.8 mUI/L (normal values: 2.8-46.1 mUI/L), and an elevated aldosterone/renin ratio (ARR) of 37.2 ng/dL/mUI/L (normal value: 3.7 (ng/dL/mUI/L). These laboratory tests were performed under conditions of normal potassium levels, a low-salt diet, and concurrent use of nifedipine and clonidine for blood pressure control. Based on these findings, the diagnosis aligned with secondary hypertension attributed to primary aldosteronism (Table 1).

**Table 1:** Evaluation of the patient’s renin-angiotensin-aldosterone axis PAC: plasma aldosterone concentration; ARR: aldosterone/renin ratio.

Blood tests	Results / Normal values
PAC (as ng/dL)	67.40 / 2.21-35.3
Plasma renin concentration (as mIU/L)	<1.8 / 2.8-46.1
ARR (ng/dL/mIU/L)	37.2 / 3.7

**Figure 1:**
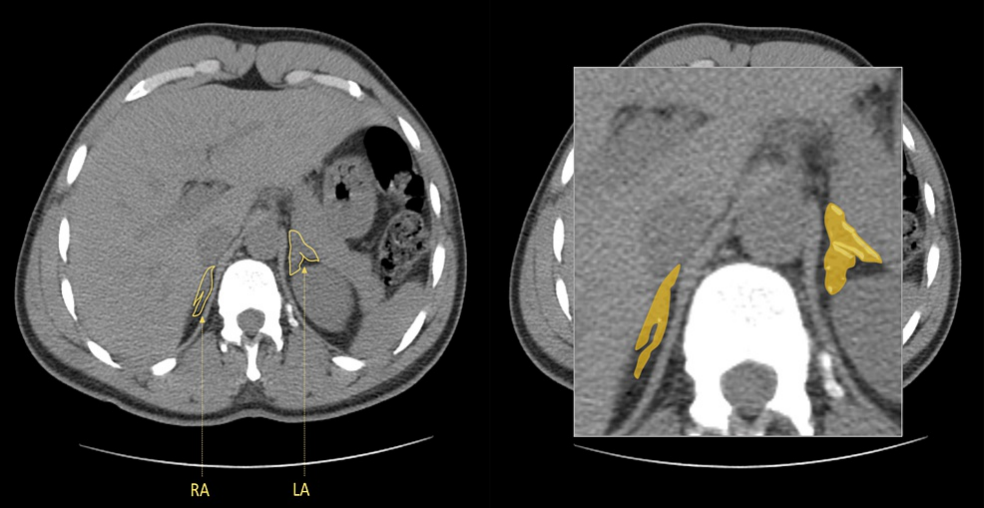
Non-contrast computed tomography scan of the abdomen and pelvis (axial view). Hyperplasia of the adrenal glands (yellow); RA- right adrenal gland; LA - left adrenal gland.

**Figure 2:**
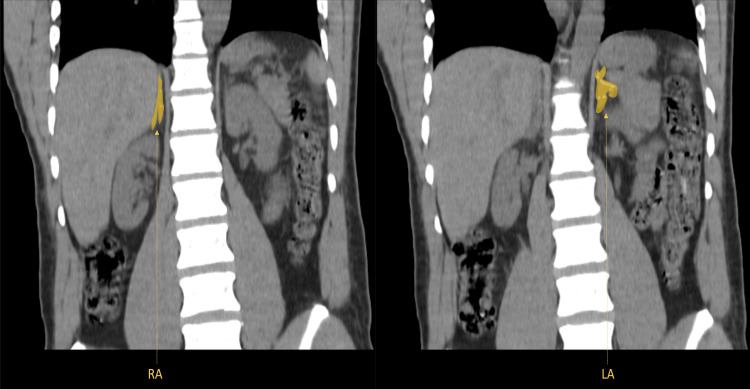
Non-contrast computed tomography scan of the abdomen and pelvis (coronal view). Adrenal hyperplasia (yellow), is characterized by severe thickening of the left adrenal gland and mild hyperplasia of the right adrenal gland; RA- right adrenal gland; LA - left adrenal gland.

**Figure 3:**
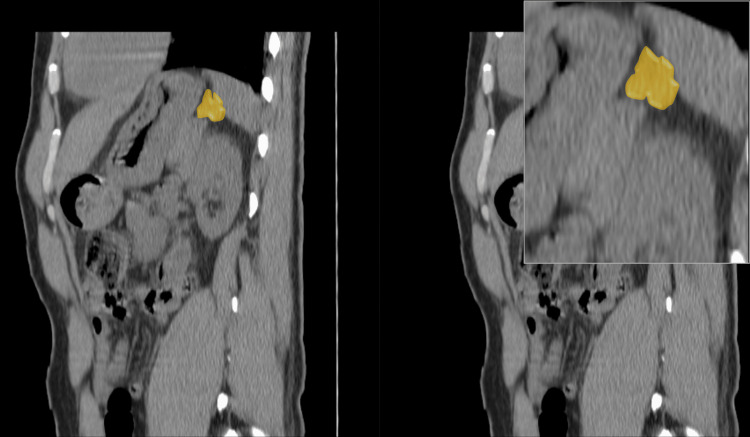
Non-contrast computed tomography scan of the abdomen and pelvis (coronal view). Severe left adrenal thickening (yellow).

Subsequently, a non-contrast CT scan of the abdomen and pelvis was performed, indicating thickening of both adrenal glands (Figures 1, 2), particularly notable in the left adrenal gland and mild hypertrophy of the right adrenal gland. No discernible nodular formations were observed (Figure 3). Regrettably, the assessment of lateralization through adrenal venous sampling was precluded due to the patient's pre-existing kidney disease. 

The diagnosis of primary aldosteronism with secondary hypertension attributed to adrenal gland hyperplasia was established, and other potential causes of secondary hypertension were also ruled out as exposed in the subsequent evaluation. We documented normal thyroid function and the absence of renal artery stenosis based on renal echo-doppler ultrasonography. Testing for pheochromocytoma and Cushing's syndrome, including plasma and urine metanephrines and cortisol levels, yielded results within the normal range. There were no signs of obstructive sleep apnea syndrome as well. Additionally, laboratory assessments disclosed hyperparathyroidism (887.10 pg/mL), hypocalcemia (8 mg/dL; albumin 40 mg/L), and anemia (Hb 9g/dL) of chronic disease with mild iron deficiency (iron 21 ug/dL; CTFF169 ug/dL; transferrin. 1.51 g/dL; ferritin 340.2 ng/mL). Autoimmune diseases were also ruled out.

To assess hypertension-induced organ damage, a transthoracic echocardiogram revealed thickening of the interventricular septum and enlargement of both atria, with a normal left ventricular ejection fraction. An ophthalmoscopic examination disclosed grade 4 hypertensive retinopathy, bilateral papilledema, and peripapillary hemorrhage.

A metabolic assessment showed a body mass index (BMI) of 26, Hb A1c level of 5.5%, and plasma lipid values as follows: cholesterol 272mg/dL, high-density lipoprotein (HDL) 48mg/dL, low-density lipoprotein (LDL) 200mg/dL, triglyceride 138mg/dL, classifying the patient as overweight with elevated cholesterol and triglyceride levels.

Regarding kidney disease, we considered it chronic based on laboratory results and ultrasound findings. Suspected causes included undiagnosed and untreated hypertension, specifically secondary to primary aldosteronism. However, the duration of disease progression remained unknown.

Throughout hospitalization, the patient's kidney function remained stable [creatinine 8.15 mg/dL; estimated glomerular filtration rate (eGFR) 7-->8 ml/min/1.73], with preserved diuresis (>0.5ml/Kg/h) and without uremic symptoms. Treatment with calcitriol, darbopoietin, and weekly intravenous iron was well-tolerated, resulting in improved levels of calcium (9 mg/dL), hemoglobin (10 g/dL), serum iron (115 mcg/dL), transferrin saturation (50.2%) and ferritin (480 ng/mL).

Given the advanced kidney disease, preparations were made for renal replacement therapy, and the patient opted for peritoneal dialysis after consultation.

Concerning hypertension, spironolactone initiation at 25 mg per day yielded no positive response, resulting in discontinuation due to hyperkalemia. Hypertension was managed with nifedipine (120 mg per day), clonidine (450 mcg per day), and carvedilol (12.5 mg per day). Statins were prescribed for cardiovascular risk prevention.

Regarding neurological deficits, positive responses were noted with blood pressure management and physical rehabilitation, evidenced by regression of the intracranial hematoma on serial cranial CT scans.

At discharge, the patient exhibited left central facial paresis and grade 4 paresis in the ipsilateral superior limb but retained independent functionality in daily activities. 

Regrettably, we were unable to conduct the follow-up ourselves due to the patient relocating to a different city. Consequently, his case was transferred to another hospital.

## Discussion

In this case study, a young Black male presented with secondary hypertension attributed to primary aldosteronism, causing extensive HMOD, including the cardiovascular and cerebrovascular systems, end-stage kidney disease, and hypertensive retinopathy.

To assess the patient's renin-angiotensin-aldosterone axis, laboratory tests were conducted under normalized potassium levels after correcting hypokalemia and following a low-sodium diet, ensuring the reliability of the renin-angiotensin-aldosterone system (RAAS) [2].

Considering potential confounding factors, the impact of clonidine, a central alpha agonist, was acknowledged. Although clonidine can suppress renin production more than aldosterone, discontinuation was not viable due to the patient's severe hypertension. Despite this limitation, we consider the obtained aldosterone/renin ratio valid, supported by the elevated aldosterone levels and other consistent findings [1-3].

Following European Society of Endocrinology guidelines, confirmatory tests such as the oral sodium loading test, captopril challenge test, and fludrocortisone oral test were deemed unnecessary, given the patient's spontaneous hypokalemia, undetectable plasma renin levels, and a plasma aldosterone concentration equal to or greater than 20 ng/dL [6]. The patient fulfilled these criteria, meeting the diagnostic criteria for primary aldosteronism [6].

Subsequent evaluation, typically involving contrast-enhanced CT imaging of the adrenal glands, was impractical due to end-stage kidney disease [3-5]. Nevertheless, radiologists noted noticeable thickening of the left adrenal gland and mild hypertrophy of the right adrenal gland. 

To confirm or exclude laterality, adrenal venous samples are recommended [7]. Unfortunately, this examination was also unfeasible due to the patient's end-stage kidney disease. Confirmation of laterality remains pending and will be considered when the patient's clinical condition permits.

The initial treatment involved mineralocorticoid antagonists as the first-line therapy [8,9]. However, a significant increase in blood potassium levels, likely attributed to end-stage kidney disease, necessitated discontinuation. Reintroduction will be considered upon the initiation of dialysis if surgical intervention is unnecessary.

The patient's condition includes cerebrovascular disease, chronic kidney disease, bilateral grade 4 hypertensive retinopathy, and echocardiographic findings of biauricular dilatation and interventricular septum hypertrophy. Undiagnosed arterial hypertension of unknown duration is believed to underlie these manifestations, emphasizing the need for early detection and prompt management of cardiovascular risk factors [1-3].

As per European Society of Cardiology guidelines, the patient carries a high risk (≥10%) of a potentially fatal cardiovascular event within a 10-year period [1].

The documented harmful effects of hyperaldosteronism include oxidative stress leading to an inflammatory state, endothelial dysfunction, and vascular remodeling. These contribute to increased afterload and arterial stiffness, adversely affecting target organs such as the heart and kidneys [1-3]. The expansion of blood volume and increased preload can cause structural damage to the heart muscle, resulting in ventricular hypertrophy, fibrosis, diastolic dysfunction, and atrial dilation. Atrial fibrillation is commonly observed in such conditions [1-3].

These findings elucidate why patients with primary aldosteronism experience a higher incidence of HMOD and cardiovascular complications compared to individuals with essential arterial hypertension of similar severity [1-3].

## Conclusions

Primary Aldosteronism is the leading cause of secondary arterial hypertension and is associated with numerous detrimental effects on the heart, arterial walls, and kidneys. This case highlights the harmful consequences of primary aldosteronism, emphasizing the necessity for early detection of arterial hypertension and other cardiovascular risk factors, as well as timely screening for secondary hypertension when warranted.
